# Seasonal Dynamics of Sand Flies (Diptera, Pshycodidae), Vectors of Cutaneous Leishmaniasis, in the City of Fez, Northern Morocco

**DOI:** 10.1155/2022/4095129

**Published:** 2022-09-07

**Authors:** Najoua Darkaoui, Abdellatif Janati Idrissi, Fatima Zahra Talbi, Youness El Fattouhi, Hajar El Omari, Mohamed Najy, Abdelkarim Taam, Abdellatif Alami, Fouad El-Akhal, Abdelhakim El Ouali Lalami

**Affiliations:** ^1^Sidi Mohamed Ben Abdellah University, Faculty of Sciences Dhar El Mahraz, Laboratory of Biotechnology, Conservation and Valorization of Naturals Resources (LBCVNR), Fez 30000, Morocco; ^2^Hassan First University of Settat, Faculty of Sciences and Technologies, Laboratory of Biochemistry, Neurosciences, Natural Resources and Environment, P.O. Box 577, Settat 26000, Morocco; ^3^National Education, Preschool and Sports, Regional Academy of Education and Formation of Fez-Meknes, Qualifying Secondary Education, Moulay Ismail High School, Meknes, Morocco; ^4^Natural Resources and Sustainable Development Laboratory, University Ibn Tofail, Faculty of Sciences, BP133, Kenitra 14000, Morocco; ^5^Laboratory of Engineering Sciences, National School of Applied Sciences (ENSA), Ibn Tofail University, Kenitra, Morocco; ^6^Laboratory of Applied Organic Chemistry, Faculty of Science and Technology, Sidi Mohamed Ben Abdellah University, Fez, Morocco; ^7^Institute of Nursing Professions and Health Techniques of Tetouan (Annex Al Hociema), Regional Health Directorate, Mohammed V Hospital, Al Hociema 32000, Morocco; ^8^Higher Institute of Nursing Professions and Health Techniques of Fez, Regional Health Directorate Fez-Meknes, EL Ghassani Hospital, Fez 30000, Morocco

## Abstract

The infections transmitted by sand flies (Diptera, Psychodidae) pose always a real health problem due to the increasing number of cases detected each year and the annual emergence of new leishmaniasis outbreaks. This study evaluated the temporal evolution of six species of sand flies in five stations inventoried between May 2017 and April 2018, in order to determine for the first time the extent of cutaneous leishmaniasis (CL) transmission in the city of Fez. The monthly impact of Fez sand fly density has been studied using all multivariate statistical analyses, including multiple factor correspondence analysis (MCA), which were performed using XLSTAT and the version of SPSS 20.0 test. Differences between concentrations were considered significant if *P* < 0.05. To better study the results obtained, different ecological indices have been studied. This study showed that these vectors developed in different sectors of the city of Fez. A total of 816 sand flies were collected from five stations in the city, belonging to three species of the genus *Phlebotomus* (46.82%) and three species of the genus *Sergentomyia* (53.18%). The seasonal fluctuation of the average density followed a bimodal evolution for the three stations Dhar Richa, Ain Nokbi, and Boujloud. The stations of Ain Nokbi (0.87 ph/m^2^) and Dhar Richa (0.467 ph/m^2^) exposed the sites to a high average density with a maximum peak during August (1.965 ph/m^2^) and July (1.87 Ph/m^2^), respectively. *S. minuta* (44.24%), *Ph. sergenti* (26.96%), *Ph. perniciosus* (10.78%), and *Ph. papatasi* (9.07%) were the most qualified species. The calculated *P* value is above the 5% significance level, so the relative abundance of these species between study sites shows no significant difference. The period of phlebotomy activity of the genus *Phlebotomus* in Fez lasts seven months from May to November with a bimodal or trimodal evolution and varies according to the species or the surveyed station. The seasonal fluctuation of sand flies could be conditioned by climatic factors where the period of activity of the species coincides with the hot months (May, June, July, and August). We have observed that the temperature factor favors the prevalence of sand flies, while the difference in the relative abundance of species between the sites is related to the difference in the bioecological conditions of each site.

## 1. Introduction

Sand flies (Diptera, Psychodidae) play a role in the transmission of leishmaniasis through the parasite *Leishmania* (Kinetoplastida: Trypanosomatidae) [1–3]. This sand fly is well adapted to tropical and subtropical climates and is highly responsive in the Mediterranean region [4, 5]. Leishmaniasis is an extremely diverse disease both clinically and epidemiologically. Thus, there are three main groups, namely visceral leishmaniasis (VL), mucocutaneous leishmaniasis (MCL), and cutaneous leishmaniasis (CL). According to the WHO, the burden of leishmaniasis remains considerable throughout the world. 98 countries and territories are endemic for leishmaniasis in 2020, including 71 countries that are endemic for VL and CL, 8 countries that are endemic for VL only, and 19 countries that are endemic for CL. As well as 7 countries reported more than 5,000 cases of CL (Afghanistan, Algeria, Brazil, Colombia, Iraq, Pakistan, and the Syrian Arab Republic), which account for 80% of the reported global incidence of CL [6–8].

In Morocco, sand flies and their geographical distribution have been studied by several authors since the beginning of the century. After the first partial analyses by Delanoë [9], Ristorcelli published four successive notes on the collections made by Langeron in the south and east of Morocco [10, 11]. In 1947, Gaud made the first study of collections from more varied regions of Morocco, where he demonstrated for the first time the presence and abundance of certain species of sand flies in Atlantic Morocco [12]. The work of Bailly-Choumara et al. [13] developed a geographical and bioclimatic synthesis of sand flies in Morocco. Based mainly on the trapping of sand flies along three meridian transects from the Rif to the Sahara, the missions of Rioux's team in different regions of Morocco have made it possible to establish a sand fly inventory [14]. In Morocco, three climatic parameters seem fundamental to explain the biogeographic distribution of the species. Indeed, bioclimatic stages explain the distribution of some species. Thus, *Phlebotomus perniciosus* extends not only in the humid and subhumid stages [15] but also in the semi-arid stages [16]. These species are suspected to be vectors of *L. infantum* [17] and have been collected in the mountains while they were absent in the lowlands. On the contrary, *Phlebotomus papatasi* was found in the arid and Saharan stage [18]. *Ph. longicuspis,* which has been shown to be a vector of *L. infantum* [19], was collected at all altitudes but with a high density between 600 and 799 m altitude. *Phlebotomus sergenti* was abundant in the semi-arid bioclimatic stage and in areas bordering the arid region [18] and widespread at altitudes between 800 and 900 m. Several studies conducted in Morocco have shown the presence of 22 species of sand flies, of which 13 belong to the genus *Phlebotomus* and 9 to the genus *Sergentomyia* [19]. However, five species threaten public health in Morocco: *Phlebotomus sergenti*, vector of *L. tropica*; *Phlebotomus papatasi*, vector of *L. major*; and *Phlebotomus perniciosus*, *Phlebotomus ariasi*, and *Phlebotomus longicuspis*, vectors of *L. infantum* [20–23]. The parasitic cycle of leishmaniasis depends on three main actors, namely the transmission vector, the pathogenic parasite, and the host. The dynamics of this cycle are conditioned by climatic and socioeconomic factors. Indeed, several studies have confirmed correlations between climatic factors and the distribution of leishmaniasis cases [24, 25].

In this article, we present for the first time the seasonal dynamics of sand flies (Diptera, Pshycodidae), vectors of cutaneous leishmaniasis, in the city of Fez, Northern Morocco by using multivariate statistical analyses. This study would be of great benefit to health services and control programmes.

## 2. Materials and Methods

### 2.1. Study Area

Fez city (Latitude: 34°03′00″ and Longitude: 4°58′59″) is located in the center north of Morocco, 180 km east of Rabat, between the Rif massif and the Middle Atlas, at an altitude of 406 m. Fez is the 2nd largest city in Morocco and covers 332 km^2^, with a population of 1,150,131 inhabitants (hab) and a density of 3464 hab/km^2^ according to the 2015 census [26]. It represents the cultural and spiritual capital of the country, is one of the most visited tourist destinations in Africa, and has been listed as a UNESCO World Heritage Site since 1981 [27, 28].

The climate of Fez is Mediterranean mixed with continental and located in the Saïs plain between the Rif in the north and the Middle Atlas in the south and suffers the effect of the mountains. At the edge of the Middle Atlas, the region of Fez is an invitation to discover the great diversity of landscapes, green valleys, cedar forests, and thermal springs [27]. The annual rainfall is between 500 and 700 mm with the presence of two distinct periods: one rainy from October to April, and another dry from June to September with a mild winter even cold and wet [29].

### 2.2. Collection of Sand Flies

Different environments were prospected. The choice of stations was based on the number of cases of leishmaniasis reported in the region from 2016 to 2017 and the state of hygiene of the various foci, including the proximity of uncontrolled public dumps, manure, presence of ruined houses, caves, cracks in the walls, stables, etc. These criteria of choice allowed us to select five stations: Ain Nokbi (34.06441.N-004.95753.W), Dhar Richa (34.07130.N-004.98241.W), Boujloud (34.06378.N-004.98537.W), Cotef (34.06650.N-004.98230.W), and Zlilig (33.96166.N-005.08804.W) (Table 1).

The sand flies were captured between May 2017 and April 2018 at the stations studied twice a month. The collection of adult specimens from different stations was based on the use of sticky-type traps (8 × 3 lodgings for each station); these are sheets of white paper format (21 × 29.7 cm) largely soaked in castor oil which has the advantages of not being repellent, being very viscous to engulf the sand flies, and being soluble in alcohol which facilitates the subsequent recovery of insects. They are either rolled into cones and inserted into the interstices of the walls or placed upright in strings before dusk and collected the next morning around 8 am. The number of trap sets was 24 per station, but the number recovered is always lower in case of loss either by the wind or voluntarily by the local population, and the trapping was carried out in the same places during the whole study period.

### 2.3. Dissection and Identification of Sand Flies

The collected specimens were washed with distilled water and then stored in 70% alcohol and cleared with 10% potash for 4 hours and with Marc-André solution for 2 hours [30]. Identification was made by examining the morphology of the male (cibarium armature, penile valve shape, number and position of spines on the genitalia, and number of apical setae on the coxite) and female genitalia (spermathecae shape, pharyngeal armature, and shape and number of cibarium teeth) [31].

### 2.4. Processing of Results

To better study the results obtained, we used the following ecological indices:

  Specific richness equals the total number of species recorded in the different stations surveyed during the study period
  Sex ratio represents the number of males to the number of females
  Relative abundance is the ratio of the number of individuals of a given species trapped to the total number of individuals in the stand
  RA = *Ni*/*N* × 100, where *Ni* is the number of individuals of species *i* and *N* is the total number of sand flies collected
  Density expressed by the number of individuals of the species captured per trap area (m^2^) and per night of capture (trapped area = trap area × number of traps used × 2)

All multivariate statistical analyses, including multiple correspondence factor analysis (MCA) and hierarchical ascending classification, were performed using XLSTAT and SPSS 20.0 trial versions. Differences between concentrations were considered significant if *P* < 0.05.

A significance level of 0.05 indicates a 5% risk of incorrectly concluding that there is a difference. A value of *P* ≤ *α* indicates that the differences between certain means are statistically significant. That is, you can reject the null hypothesis and conclude that not all population means (species of sand flies) are equal.

Factorial analysis of correspondences (AFC) was carried out for the set of variables selected (relativity of abundance of the species studied), making it possible to identify the classification of groups on the factorial plan, the axis F1 which brings the statistical information the largest in the AFC compared to the F2 axis.

Hierarchical ascending classification (AHC) is performed. It is sought that the individuals grouped within the same class are as similar as possible while the classes are the most dissimilar.

The principle is to bring together individuals according to a criterion of resemblance defined beforehand which will be expressed in the form of a matrix of distances, expressing the distance existing between each individual taken two by two.

Multiple correspondence analysis is for qualitative variables. It makes it possible to arrive at representation maps on which one can visually observe the proximities between the categories of qualitative variables (density of species) and the observations (study sites).

## 3. Results

### 3.1. Taxonomic Diversity of Sand Flies

A total of 816 specimens were collected in the five stations. Six species of sand flies (3 species of *Phlebotomus* genus and 3 species of *Sergentomyia* genus) were identified in the city of Fez. Thereby, the *Phlebotomus* and *Sergentomyia* represented 46.82% and 53.18%, respectively, with a sex ratio of 20.47. *S. minuta* (44.24%) and *Ph. sergenti* (26.96%) were the most abundant species, followed by *Ph. perniciosus* (10.78%). *Ph. papatasi* (9.07%), *S. fallax* (7.97%), and *S. antennata* were rare and represented only 0.98% of the collected flies.

According to the spatial distribution, the sand flies were present with 37.87% in Ain Nokbi, 18% in Boujloud, 19.68% in Dhar Richa, 16.23% in Zlilig, and 8.58% in Cotef (Table 2) The stations Zlilig and Dhar Richa represent high-risk stations for leishmaniasis, given the number of specimens of medical interest collected.

### 3.2. Density of Sand Flies Collected from Different Stations in the City of Fez

**Figure 1:**
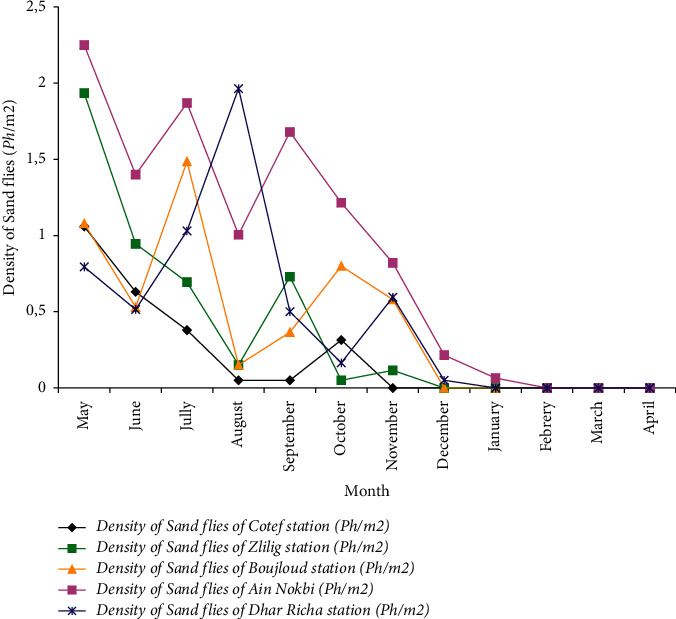
Seasonal activity of sand flies in different surveyed stations of the city of Fez from May 2017 to April 2018.

In terms of average density, the monthly evolution of sand fly density varied from station to station and was spread over a nine-month period from May 2017 to January 2018 (Figure 1). The stations of Ain Nokbi (0.87 ph/m^2^) and Dhar Richa (0.467 ph/m^2^) represented the sites with a high average density, followed by Boujloud (0.415 ph/m^2^), Zlilig (0.385 ph/m^2^), and Cotef (0.20 ph/m^2^). The seasonal fluctuation of the average density followed a bimodal evolution for the three stations Dhar Richa, Ain Nokbi, and Boujloud with a maximum peak, respectively, during August (1.965 ph/m^2^) and July (1.87 ph/m^2^; 1.48 ph/m^2^).

### 3.3. Seasonal Fluctuation of Sand Flies

Calculating the density of sand flies for twelve months allowed us to study the seasonal dynamics of different species. The period of activity of sand flies varies according to the genus. Indeed, the sand flies of medical interest belonging to the *Phlebotomus* genus (2.12 ph/m^2^) are active for seven months from May to November. On the other hand, the species of *Sergentomyia* genus (2.41 ph/m^2^) present a seasonal activity that lasts up to nine months (from May 2016 to January 2017), driven by *S. minuta*.

**Figure 2:**
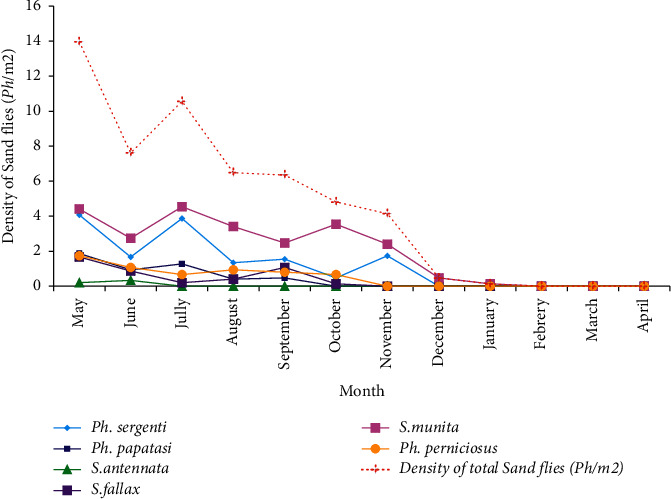
Seasonal dynamics of different specimens of sand flies captured from different stations in Fez city from May 2017 to April 2018.

For the *Phlebotomus* genus, in relation to the total number of specimens captured, *Ph. sergenti* species were the most dominant. They can be spread over seven months on average (from May to November). They present a trimodal distribution with a maximum peak during July (3, 873 ph/m^2^). *Ph. perniciosus* comes in the second position; its seasonal activity is spread over a period of six months and cannot tolerate the months beyond October. Its dynamics follow a diphasic distribution with two distinct peaks in August (0.935 ph/m^2^) and October (0.67 ph/m^2^). *Ph. papatasi* is present only at Zlilig and Dhar Richa, and active only five months on average. They followed a bimodal dynamic with a maximum peak in July (1.27 ph/m^2^) (Figure 2).

### 3.4. Statistical Study

**Figure 3:**
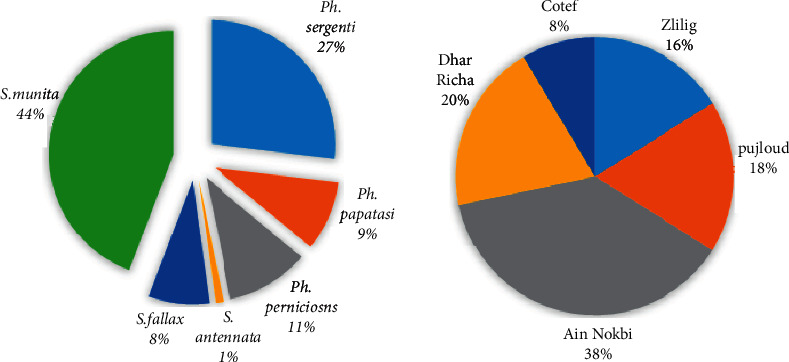
Relative abundance of sand fly species collected in the five stations of the city of Fez from May 2016 to April 2017.

The relative abundance of the species collected in relation to all sites surveyed as well as to each site surveyed is shown in Figure 3.

The prevalence of species differs from one site to another; *S. minuta* and *Ph. sergenti* are in the majority with a cumulative rate for these two species of 30.39% at Ain Nokbi, 12.62% at Dhar Richa, 8.46% at Zlilig, 5.02% at Cotef, and 14.71% at Boujloud.

**Figure 4:**
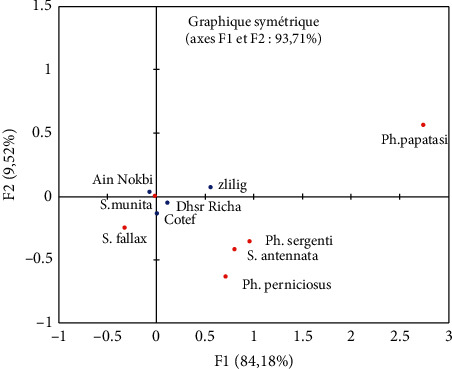
Axis factorial map (F1-F2, 93.71%) of the sand fly species inventoried in the six study stations (CFA).

To highlight the differences in the distribution of sand fly species within the different study stations, the entomological data were processed by factorial correspondence analysis and hierarchical ascending classification (Figure 4).

F1 and F2 represent the maximum relative abundance results of the species collected in the five stations. Since the calculated *P*-value is above the 5% significance level, the relative abundance of these species between the study sites does not show significant differences.

**Figure 5:**
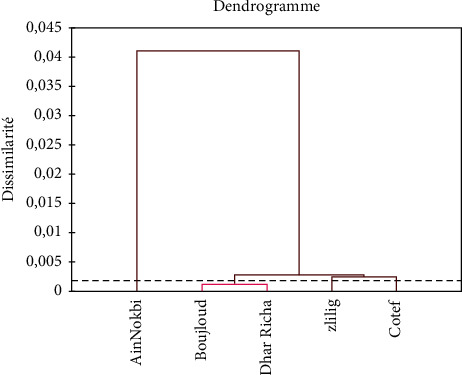
Dendrogram of the classification of the study sites.

The ascending hierarchical classification revealed three classes of species: C1 (*Ph. sergenti*), C2 (*Ph. papatasi*, *S. fallax*, *Ph. perniciosus*, and *S. antennata*), and C3 (*S. minuta*). However, the projection of the study stations on the two factorial plans of the ACM (Figure 4) allows us to make a classification constituted of four well-distinguished groups. This is well confirmed in Figure 5.

The first group corresponds to the station of Zlilig which absorbs 16% of the species inventoried, the second is formed by two stations, namely Boujloud and Dhar Richa, the third includes the station of Ain Nokbi with 38% of species collected, and the last axis is formed by Cotef.

**Figure 6:**
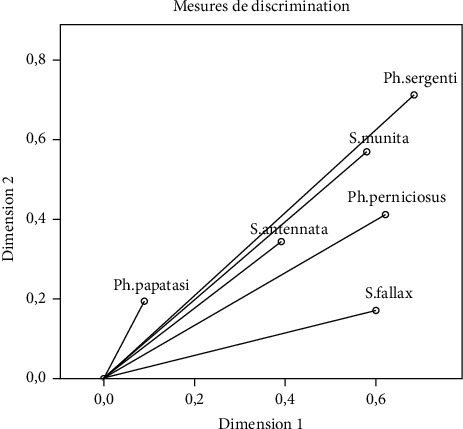
Discrimination measures of the studied variables (density of species studied).

To see the most relevant variables (density of species studied) in the study period, a measure of discrimination was designed. The density of the species (*Ph. sergenti*, *S. minuta*, and *Ph. perniciosus*) is very essential than the others as shown in Figure 6. Each vector measures the languor density of the species found.

The months constituting the rainy season have been less characterized by the density of sand flies, while the majority of the species have been found in the part where the months are very hot (May, June, July, and August).

This allows us to say that the temperature factor could favor the prevalence of sand fly species.

## 4. Discussion

The genus *Phlebotomus* includes 7 subgenera and 33 species, 5 of which have already been reported in Morocco, and includes all species involved in the transmission of leishmaniasis in the Old World. In the city of Fez, the faunistic inventory of sand flies revealed a total of six species, three of which belonged to the genus *Phlebotomus* recognized as being of medical interest. The first was *Ph. sergenti* (26.96%), which is the most dominant in Fez. According to the literature, it is confirmed as a vector of *L. tropica* in North Africa, Middle East, and Central Asia [32, 33]. This species is widely distributed in arid and semi-arid areas and its period of activity varies from six to seven months (between April and November) in subhumid bioclimates [13, 34, 35]. The second species was *Ph. perniciosus* (10.79%), a proven vector of canine and human visceral leishmaniasis [36, 37], followed by *Ph. papatasi* (9.07%), a proven vector of *L. major* [38, 39]. For the *Sergentomyia* genus, *S. minuta* was the most dominant species in Fez with a proportion of 44.24%, followed by *S. fallax* (7.97%) and *S. antennata* (0.98%).

The result of our study area is similar to the Province of Sefrou [21], Province Moulay Yacoub [40], Province Boulemane [18], and Province Meknes [41].

In terms of seasonal dynamics, the *Phlebotomus* genus in the city of Fez knows a period of phlebotomine activity that lasts seven months from May to November with a bimodal or trimodal evolution and which varies according to the species or station surveyed. This result is confirmed by different studies made in the Mediterranean region [42], Sefrou Province [21, 22], and Meknes [41].

According to the species, *Ph. sergenti* followed a triphasic evolution with a first peak in May. This result corroborates with that obtained by Idrissi et al. in 1997 [43]. The same species was collected in Taza, a semi-arid area in northern Morocco, with seasonal activity from June to November, but with only two distinct peaks, the first in August and the second in October [44]. The same period of activity has been reported in Morocco, in Chichaoua (Imintanoute), with a single peak in August [45]. However, in Marrakech, this species was only active from April to June [46]. The presence of *Phlebotomus sergenti*, the vector of cutaneous leishmaniasis caused by *L. tropica*, in our sample confirms the possibility of local transmission of this parasite. This species encountered in the five capture sites was intradomiciliary, which could provide *Ph. sergenti* with an endo-anthropophilic character.

*Ph. perniciosus*, the species most often collected in the subhumid, semi-arid, and arid stages, is in the third place. It is captured in and near houses [45, 47]. In Italy [48], *Ph. perniciosus* appears quite early in the year, generally from May, and persists until October. Its prevalence is significantly higher in domestic sites than in wild resting sites [48]. In addition, Rossi et al. in 2008 captured a significant number of *Ph. perniciosus* in sites where avian or ovine influenza was reported; for these authors, this may imply the involvement of the species *Ph. perniciosus* in virus transmission.

*Ph. papatasi* is one of the most studied species because of its frequency and the importance of its geographical distribution area; it is collected throughout the country where it is one of the most abundant species, and its role as a vector of CL due to *L. major* is confirmed in Morocco and sub-Saharan Africa and its presence is more important in North Africa, the Mediterranean, and Asia [7]. In this region, *Ph. papatasi* shows two density peaks which corresponds to other works with two peaks in autumn and summer but with a difference in dominance in crops and its activity throughout the year [49].

This species has no diapause in the region of Marrakech, the population peaked in June and November, and its preferred temperature varied between 32 and 36°C but no significant correlation was found between its density and temperature [46]. Despite the presence of *Ph. papatasi*, and its long period of activity, the city of Fez is far from an unpredictable outbreak of zoonotic cutaneous leishmaniasis due to *L. major* thanks to the absence of gerbil (*Meriones shawi*) as a reservoir host.

The population density of *Phlebotomus* species was relatively very low compared with the density of species of the genus *Sergentomyia*, and this is the case of another study [50], which confirms that *Sergentomyia* is generally rich in species and numbers, but in Africa south of the Sahara and Southeast Asia often overwhelm *Phlebotomus* species [50].

Phlebotomines of the genus *Sergentomyia* have long been known to feed generally on reptiles and have been implicated in the transmission of *Sauroleishmania* (Trypanosomatidae) from reptiles in the Old World, although some species may feed on humans [49]. Several viruses have been isolated from sand flies of the genus *Sergentomyia*, such as Saboya virus and ArD 95737 and ArD111740 viruses; recently, the isolation of RNA of human viruses in species of the genus *Sergentomyia* has allowed to suspect their role as vectors [49]. At the level of Fez, this study showed that these species were well represented by the species *S. minuta*, with a high density and sex ratio in favor of males. This result corroborates with the work of Boussaa [46] except for of *S. fallax* which had a sex ratio in favor of females and *S. antennata* which was absent in their collections.

Seasonal fluctuations of sand flies could be conditioned by climatic factors, and some studies have shown that they are well related to bioclimatic affinities [51]. In Fez, the period of activity of sand flies, especially of medical interest, from May to November coincides with the dry period of the year. According to the role of climatic factors, the temperature could favor the multiplication of sand flies, increasing their frequency and reinforcing their vectorial capacity and thus facilitating the transmission of leishmaniasis to different hosts or reservoirs [21, 52, 53].

## 5. Conclusion

This entomological study at Fez demonstrated that the seasonal fluctuations of our captured species and their period of activity from May to November coincide with the dry period of the year, which is characterized by an increase in temperature and a decrease in precipitation which allows us to say that the temperature factor could favor the prevalence of sand fly species. Thus, the difference in the relative abundance of species between the sites is linked to the bioecological conditions of these sites. The presence of *Ph. sergenti*, a vector of CL caused by *L. tropica*, in our sample confirms the possibility of local transmission of this parasitosis. The results of this detailed study on the temporal distribution of different species of medical interest provide important reference data for the planning of control interventions at the time of peaks in density and allow the control of disease transmission in the event of epidemics.

To better understand the ecology and biology of the vector, different axes, namely the analysis of the characteristics of the potential breeding sites of sand flies and the study of trophic preference, will be of great value to accomplish this work.

## Figures and Tables

**Table 1:** Environmental conditions of the five-trapping station.

station	Geographical coordinates	Nature of biotope	State of hygiene	Activity in the vicinity
Ain Nokbi	34.06441.N-004.95753.W	Destroyed market	Waste of all kinds	Stable and houses
Dhar Richa	34.07130.N-004.98241.W	Caves and stables	Presence of manure and waste of any kind	Stable and houses
Boujloud	34.06378.N-004.98537.W	Wall and caves	Organic waste	Commercial activity
Cotef	34.06650.N-004.98230.W	Old textile complex	Industrial and organic waste	Industrial and residential areas
Zlilig	33.96166.N-005.08804.W	Caves and rural area	Manure and waste of any kind	Breeding

**Table 2:** Relative abundance of sand flies caught in different stations in the city of Fez, Morocco, from May 2016 to April 2017.

Stations	*Sergentomyia*						*Phlebotomus*					
	*Sa*		*Sm*		*Sf*		*Pp*		*Ppa*		*Ps*	
	F	M	F	M	F	M	F	M	F	M	F	M
Cotef	1	2	10	11	0	10	0	16	0	0	3	17
Ain Nokbi	0	3	0	216	2	38	2	16	0	0	0	32
Dhar Richa	0	0	2	45	0	0	0	33	1	24	0	56
Boujloud	0	0	7	51	1	14	0	9	0	0	0	62
Zlilig	0	2	3	16	0	0	0	12	1	48	5	45
Total	**1**	**7**	**22**	**339**	**3**	**62**	**2**	**86**	**2**	**72**	**8**	**212**

*Pp*, *Ph. perniciosus*; *Sm*, *S. minuta*; *Sf*, *S. fallax*; *Sa*, *S. antennata*; *Ppa*, *Ph. papatasi*; *Ps*, *Ph. sergenti*; F, female; M, male.

## Data Availability

All the data analyzed during the study are included with links in the article.
